# Implementation of an adaptable semi-automated nonventilator hospital-acquired pneumonia (nvHAP) surveillance system in swiss acute care hospitals: a feasibility study

**DOI:** 10.1017/ice.2026.10416

**Published:** 2026-05

**Authors:** Aline Wolfensberger, Mirjam Faes Hesse, Marc Hany, Claudine Reiber, Anna Müller, Marius Zeeb, Gioele Capoferri, Sabine Kuster, Kai Manuel Adam, Ulrike Schnee-Lach, Laila Elhilali-Keller, Sarah Tschudin-Sutter, Davide Bosetti, Etienne Chalot, Marie-Céline Zanella, Stephan Harbarth, Jasmin Männer, Fabian Grässli, André Riedel, Domenica Flury, Indrani Sen, Benedikt Wiggli, Danielle Vuichard-Gysin, Walter Zingg, the Swissnoso Group

**Affiliations:** 1 Department of Infectious Diseases and Hospital Epidemiology, University Hospital Zurich and University of Zurich, Zurich, Switzerland; 2 Institute for Implementation Science in Health Care, University of Zurich, Zurich, Switzerland; 3 Division of Infectious Diseases, University Hospital Basel, Basel, Switzerland; 4 Infection Control Programme and WHO Collaborating Centre, Geneva University Hospitals and Faculty of Medicine, Geneva, Switzerland; 5 Division of Infectious Diseases, Infection Prevention and Travel Medicine, HOCH Cantonal Hospital St.Gallen, St.Gallen, Switzerland; 6 Data Innovation Team, Cantonal Hospital Baden, Baden, Switzerland; 7 Department of Infectious Diseases & Infection Control, Cantonal Hospital Baden, Baden, Switzerland; 8 Division of Infectious Diseases and Infection Prevention, Thurgau Hospital Group, Frauenfeld and Munsterlingen, Munsterlingen, Switzerland

## Abstract

**Objective:**

nvHAP (nonventilator hospital-acquired pneumonia) can affect all non-intubated patients, and semi-automated systems enable incidence surveillance. This feasibility study evaluated the performance and implementation of a semi-automated nvHAP surveillance in Swiss acute care hospitals.

**Design:**

Multicenter feasibility study

**Setting:**

Seven Swiss acute care pilot hospitals representing different sizes and language regions

**Patients:**

Inpatients of the participating hospitals.

**Methods:**

Hospitals implemented an adaptable nvHAP selection algorithm including one to five indicators (radiology procedure, radiology report, leukocytes, body temperature, intubation). Five hospitals performed manual review on the preselected patients following standardized definitions. Performance characteristics of the algorithms (i.e., sensitivity and percentage records to manually review) and time investment to implement the semi-automated surveillance were evaluated. Barriers and facilitators for implementation were identified through interviews.

**Results:**

Hospitals implemented algorithms including one, two, four and five indicators. Sensitivity ranged from 98% to 100% in larger hospitals. Percentage of records to manually review ranged from 1% to 6% in hospitals that surveyed the total patient population and was 13% in one hospital that focused on two high-risk departments. Time for technical implementation varied from 55 to 437 hours. Mean time for manual review per preselected patient was 14 minutes and decreased with experience. Implementation facilitators included preprocessed data, team experience in similar projects, and external support.

**Conclusions:**

Semi-automated nvHAP surveillance was feasible and sufficiently sensitive regardless of the algorithm. It required effort for setup and manual review. Algorithm adaptability enabled the implementation in hospitals with limited electronically available data or IT resources.

## Introduction

Pneumonia and lower respiratory tract infections (LRTI) are among the most frequent healthcare-associated infections globally.^1–3^ The prevalence of nonventilator-associated hospital-acquired pneumonia (nvHAP) is roughly double that of ventilator-associated pneumonia (VAP).^2,3^ Hospital-acquired pneumonia (HAP) in ventilated and non-ventilated patients together is responsible for most disability-adjusted life years (DALY) of all hospital-acquired infections (HAI).^4^ Mortality from nvHAP is up to 30%,^5–7^ and nvHAP generates significant hospital costs.^5,8^

Because all hospitalized patients are at risk for nvHAP, surveillance expands to the entire hospital population. The University Hospital Zurich (USZ) has developed and validated a semi-automated surveillance system for nvHAP in 2017/2018.^9^ The preselecting classification algorithm with five indicators – radiology procedure, radiology report, leukocytes, body temperature, intubation – closely mirrors the European Centre for Disease Prevention and Control (ECDC) HAP criteria,^10^ and reduced the number of patients for manual review at USZ by 93% with high sensitivity.^9^

All automated surveillance systems use routine data from electronic health records (EHR), which are increasingly being introduced in the United States^11^ and Europe.^12^ Still, some hospitals lack complete electronic records or have insufficient resources in information technology (IT) for data processing. In the USZ patient population we found that certain single indicators and selection algorithms with fewer indicators were able to decrease the number of records to manually review by >90%.^13,14^ This suggested that the USZ nvHAP preselection algorithm could be used in an adapted form in hospitals with limited datasets or limited IT-resources.

In view toward a national nvHAP surveillance in Switzerland, we conducted a feasibility study to evaluate the implementation and performance of an adaptable nvHAP selection algorithm in pilot hospitals. In addition to sensitivity and percentage of records to manually review, the workload for technical implementation, the time for manual review of preselected records, and contextual implementation determinants of the semi-automated surveillance were assessed.

## Methods

### Study setting and patients under surveillance

For this feasibility study, seven Swiss acute care hospitals were purposefully selected. Two academic tertiary-care centers, two public general hospitals, and three community hospitals were included. One hospital was French-speaking, the others were German-speaking. The hospitals were free to choose the patient population for nvHAP-surveillance.

### Adaptable preselection algorithm and minimum data set

The USZ algorithm represented the adaptable “standard algorithm.” It included the indicators “chest radiology procedure,” “chest radiology report,” “leucocyte count,” “body temperature,” and “intubation.” “Chest radiology procedure” (type and date of procedure) was the only minimally required indicator, alongside admission and discharge dates. Patients were preselected if they had at least one “chest radiology procedure of relevance.” A radiology procedure was considered “relevant” if conducted >48 h after hospital admission (or anytime in patients who were readmitted within 10 days), and when it was in a predefined temporal relationship with leucocyte count, body temperature, and respiratory device (if this data was available) (Appendix 1). Radiology procedures were classified as non-relevant if the corresponding radiology report explicitly ruled out pneumonia, based on classification of the report according to a simple text analysis, identifying terms excluding pneumonia, i.e. “no” and “infiltrate” in one sentence, without a “restricting term” (e.g., “but”, “left”) in the same sentence (Appendix 2a). For hospitals unable to implement the standard algorithm, modified versions with less indicators were allowed. For the hospital in French-speaking Switzerland, customizing/translating the radiology text analysis was required.

### Team composition, material, training, and support

Four pilot hospital teams were established with a project lead, IT staff for technical implementation of the algorithm, and data collectors for manual review (Table 1). The community hospitals were networked with one of the public general hospitals, whose team was responsible for implementing and conducting the surveillance. The teams received all materials to implement the computerized algorithm, including a Python code, an Excel data-entry form, an analysis/reporting tool, and manuals. The Python code was designed to be configurable, allowing to include indicators that were available in the respective hospital. The IT specialist of the coordination center provided ongoing support. For manual review, hospitals were provided with a handbook and received a mandatory two-hour training session. The coordination team was available to support the hospital and provide training as needed.

**Table 1.** Pilot hospitals and team composition

	Hospital 1	Hospital 2	Hospital 3	Hospital 4	Hospital 5–7
**Type of hospital**	Academic tertiary care center	Academic tertiary care center	Public general hospital	Public general hospital	Community hospitals
**Pilot hospital team**					
Project lead	Quality manager (Senior IPC physician in background)	Senior IPC physician (supported by 2 other senior IPC physicians)	Senior IPC physician	Senior IPC physician	1)
Implementation of the computerized algorithm	1 IT expert 2 senior physicians	1 IT expert 1 senior physician	1 data manager 1 senior physician	1 IT expert 1 senior physician	1)
First manual review	2 study nurses 1 medical student	2)	1 (study) nurse 1 clinical nurse specialist in IPC	2)	1)
Second manual review	2 senior physicians (intermittently)	2)	1 junior physician, supported by 2 senior physicians	2)	1)

Abbreviations: IPC, infection prevention and control; IT, Information Technology.

1) performed by team from public general hospital 2) no manual surveillance conducted.

### Pneumonia surveillance definitions and manual review

The pneumonia definition criteria of this study aligned with the ECDC criteria for HAP.^10^ Pneumonia was defined with the presence of radiological signs, fever or elevated or reduced leucocyte counts, and clinical signs and symptoms such as cough, dyspnea, or worsening of gas exchange (Appendix 3). Pneumonia in patients who were continuously intubated during the full 48h before symptom onset were considered VAP, and thus not subject of this study. We introduced indeterminate HAP (iHAP) as a novel classification. Patients never intubated in the 48 hours before symptom onset were classified as nvHAP, whereas patients who were both intubated and extubated within the preceding 48 hours were classified as iHAP. In the following sections, nvHAP is used as an umbrella term and includes iHAP.

All preselected patients underwent manual review by a first reviewer, typically someone with a nursing background. In cases of uncertainty, a second reviewer with expertise in infectious diseases provided additional assessment.

### Performance characteristics of algorithms

Sensitivity of the algorithm was assessed using full manual surveillance as the reference standard. A sample size of 73 patients with nvHAP per hospital was estimated sufficient to demonstrate a sensitivity of 95% with a ±5% error margin. As nvHAP is a rare event, we selected a validation cohort with patients at higher probability for HAP, i.e. patients with a discharge diagnostic code for HAP (U69.0x), and - in smaller hospitals - complimented by 200 randomly selected patients with a hospital stay of at least two weeks. Sensitivity was calculated as follows:

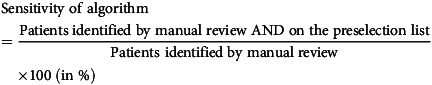




Percentage of records to manually review was calculated as follows:

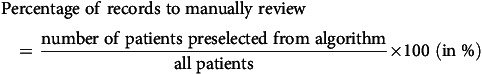




Finally, the number of patients needed to be screened from the list of preselected patients to detect one patient with nvHAP was calculated.

### Time investment and implementation determinants

All pilot hospital team members kept track of hours spent on the project. To identify determinants (i.e., barriers and facilitators) for technical implementation and manual review, two 20-minute interviews were conducted with members of the pilot teams at the start and the end of the project by a member of the coordination team (MFH) (interview guide see Appendix 4). Notes were coded deductively according to constructs of the updated consolidated framework for implementation research (CFIR).^15,16^ CFIR is a comprehensive, meta-theoretical framework including five domains (i.e., Innovation, Outer Setting, Inner Setting, Individuals, and Implementation Process) which is used to help understand the factors that influence how well an innovation is implemented in real-world settings. Deductive coding was supplemented by additional inductively created codes to capture nuances in the data.

### Ethics

The project was a quality improvement project and the necessity for formal ethical evaluation was waived from all responsible cantonal ethical committees (BASEC Req-2023-00148). All individual patient data remained in the pilot hospitals; only aggregate data were reported to the coordination center.

## Results

The 5-indicator standard algorithm was implemented in one hospital (hospital 1); the other six hospitals implemented adapted algorithms, with four, two or one indicator (Table 2). One hospital included only patients from two departments with patients at intrinsically higher risk for nvHAP, while the other six hospitals included all hospitalized patients (none served psychiatric, and only one (hospital 4) served pediatric patients). All hospitals included patients discharged between the months of January and June 2023. The German-speaking hospitals used the text-analysis provided by the coordination center, while the hospital in French-speaking Switzerland translated the text-analysis into French (Appendix 2b). Manual patient review was conducted in five hospitals by two teams, i.e. one team from the public general hospital and its associated network of three community hospitals, and another team from a tertiary care center.

**Table 2.** Components and performance characteristics of algorithms per hospital

	Hospital 1	Hospital 2	Hospital 3	Hospital 4	Hospital 5	Hospital 6	Hospital 7
Hospital type	Academic tertiary care center	Academic tertiary care center	Public general hospital	Public general hospital	Community hospital	Community hospital	Community hospital
Hospital beds (thereof ICU beds)	748 (40)	2,083 (32)	685 (36)	400 (12)	195 (7)	90 (0)	96 (0)
Patient population under surveillance	All	Patients of two high-risk departments (no ICU)	All	All	All	All	All
**Indicators included of the algorithm**							
Chest Radiology procedure	X	X	X	X	X	X	X
Chest Radiology report	X	X	X	X	–	X	X
Leucocyte counts	X	X	–	X	–	X	X
Temperature	X	X	–	X	–	X	X
Intubation data	X	–	–	–	–	–	–
**Percent records to manually review** (95% CI)	4 (4-4)	13 (12-14)	6 (6-7)	4 (4-5)	5 (4-6)	1 (1-1)	3 (2-3)
Number of observed patients	20,924	8,521	17,561	22,927	5,662	3,070	2,620
Number of preselected patients	812	110	1,109	998	282	29	70
**Number of patients needed to review for one nvHAP**	6.6 (812/123)		8.8 (1120/127)		18.8 (282/15)	7.3 (29/4)	10 (70/7)
Number of patient with nvHAP or iHAP	123		127		15	4	7
Number of preselected patients	812		1120		282	29	70
**Validation sample**							
Patients with ICD-10 HAP	188	250	183	130	11	23	33
Patients with >2weeks hospital stay (randomly selected)					200	200	200
Of validation sample: patients fulfilling nvHAP or iHAP definitions according to manual review	48	64	34	27	4	4	3
**Sensitivity of algorithm** (in %, 95% CI) (No. patients with nvHAP in validation sample flagged by algorithm/No. patients with nvHAP in validation sample)	100 (93–100) (48/48)	98.4 (92–100) (63/64)	100 (90–100) (34/34)	100 (87–100) (27/27)	100 (40–100) (4/4)	75 (19–99) (3/4)	100 (29–100) (3/3)

Abbreviations: CI, confidence interval; HAP, hospital-acquired pneumonia; ICD, International Statistical Classification of Diseases and Related Health Problems; X, indicator included in algorithm.

### Performance characteristics of the selection algorithms

Table 2 summarizes the performance characteristics of the algorithms. Sensitivity of the algorithms implemented in the larger pilot hospitals (No. 1–4) – including 2, 4 and 5 indicators - ranged from 98.4% (95% CI: 91.6–100) to 100% (95% CI: 92.6–100). Due to the small number of patients in the validation sample of the community hospitals (Hospitals 5–7), precise quantification of sensitivity was not possible.

Percentage of records to manually review was highest, i.e. 13% (95% CI: 12.2–13.6), in the hospital that applied the algorithm on patients from two high-risk departments. It was lowest, i.e. 1% (95% CI: 0.6–1.4), in a community hospital that applied a 4-indicator algorithm on all hospitalized patients. The mean number of patients needed to be manually reviewed to detect one patient with nvHAP from the list of preselected patients ranged from 6.6 to 18.8.

### Time investment

Table 3 summarizes time spent by pilot hospital and task. To technically implement the algorithm, a mean of 198 hours (from 55 to 437 hours) was spent, including tasks like data extraction and processing, data verification, implementation of the Python code, testing the algorithm performance, and iterative improvement steps. In all hospitals, validating the surveillance based on a manually reviewed patient cohort revealed the necessity to check back input data. Among the two pilot hospital teams conducting manual nvHAP review, the first reviewer spent a mean of 11.2 minutes and the second reviewer a mean 2.3 minutes per preselected patient.

**Table 3.** Time investment

		Hospital 1	Hospital 2	Hospital 3	Hospital 4	Hospitals 5–7	All hospitals (mean)
General tasks/preparatory steps	Cum. hours	***Total: 26*** IT expert: 3 Sen. phys.: 4.5 IP nurse: 4 Qual. man.: 14.5	***Total: 20.5*** IT expert: 2.5 Sen. phys.: 13 Jun. phys.: 5	***Total: 47.5*** Sen. phys.: 11 Jun. phys.: 4 IP nurse: 11 Proj. coord.: 21.5	***Total: 15.5*** IT expert: 1 Sen. phys.: 14.5	1)	*Mean all hospitals:* *273 hours*
Implementation of Algorithm	Cum. hours	***Total: 55*** IT expert: 46 Qual. man.: 9	***Total: 437*** IT expert: 356 Jun. phys.: 52 Sen. phys.: 29	***Total: 121*** IT expert: 119 Sen. phys.: 2	***Total: 178*** IT expert: 178	1)	*Mean all hospitals:* *198 hours*
Mandatory training sessions	Cum. hours	***Total: 16*** Jun. phys.: 8 IP nurse: 8	n.a.	***Total: 6*** Jun. phys.: 2 IP nurse: 4	n.a.	1)	*Mean all hospitals:* *9 hours*
	Hours per pers.	Jun. phys.: 4 IP Nurse: 4	n.a.	Jun. phys.: 2 IP nurse: 2	n.a.	1)	
Additional training sessions	Cum. hours	***Total: 5*** Qual. man.: 1 IP nurse: 3 Med. stud.: 1	n.a.	***Total: 12*** IP nurse: 12	n.a.	1)	*Mean all hospitals: 11 hours*
	Hours per pers.	Qual. man.: 1 IP nurse: 3 Med. stud.: 1	n.a.	IP nurse: 6	n.a.	1)	
Manual nvHAP Surveillance (6 months)	Cum. hours	***Total: 146*** IP nurse (1st look): 32 Med. stud. (1st look): 80 Jun. phys. (2nd look): 34	n.a.	***Total: 400*** IP nurse (1st look): 348.5 Jun. phys. (2nd look): 36.5 Sen. phys. (2nd look): 15	n.a.	1)	*Mean all hospitals: 273 hours*
	Minutes per patients on list	1st look: 8.3min 2nd look: 2.5min	n.a.	1st look: 14.1min 2nd look: 2.1min	n.a.	1)	Mean all hospitals: 14 min/patient on list

Caption: Time investment per professional group, project task and hospital. Cumulative hours are hours in total spent by each professional group and the team. Hours per person are mean hours per professional group and project task, relevant in hospitals that made the decision to involve more than one team member per professional group. Minutes per patients on list are mean minutes spent to manually review one preselected patient, divided in first reviewer (usually with nursing background) and – if necessary – second reviewer (usually an infectious disease physician).

Abbreviations: Cum. Hours, Cumulative hours; IP nurse, infection prevention nurse; IT expert, Information technology expert; Jun. phys., junior physician; Med. stud, medical student; min, minutes; per pers., per person; Proj. coord., project coordinator; Qual. man., quality manager; Sen. phys.; senior physician.

### Determinants of implementation success

Twenty-five interviews were conducted, 13 at baseline and 12 at the end of the project. A total of 232 note segments were coded. Barriers and facilitators for adoption and implementation of the surveillance system are listed in Table 4.

**Table 4.** Determinants (barriers and facilitators) for adoption, implementation and conduct of surveillance

CFIR Domain	Determinants
Inner setting and individuals (pilot hospital team and its immediate work environment)	Facilitators Alignment with IPC and quality management goals Strong interprofessional collaboration IT- and IPC-Team experience in similar surveillance projects Team members experienced in manual reviewing Barriers Ambiguous responsibilities and lack of onboarding Lack of human resources
Outer setting (i.e.,other hospital departments, hospital management, IT infrastructure, and the broader healthcare environment)	Facilitators Project alignment with hospital goals Well-established data warehouse with preprocessed data Barriers Complex EHR systems, more than one EHR needed for manual review
Innovation (semi-automated nvHAP surveillance system, including the computerized algorithm, materials, and the support)	Facilitators Coordination center IT and manual review support Well-designed project materials Barriers Manual review part of the semi-automated surveillance, specifically time needed for manual review
Implementation process	Facilitators Teamwork, task allocation, and milestone planning Close collaboration between IT specialists and healthcare professionals Engagement with external experts Meticulous planning

Abbreviations: CFIR, Consolidated framework for implementation research; EHR, electronic health record; IPC, infection prevention and control; IT, information technology; nvHAP, nonventilator hospital acquired pneumonia.

Within the *inner setting* domain, defined as the pilot hospital team and its immediate work environment, the alignment with IPC and quality management goals, and the strong interprofessional collaboration facilitated implementation. Teams with prior experience in similar projects had an advantage, while staff turnover, insufficient onboarding, and ambiguous responsibilities were implementation barriers. A lack of human resources prevented manual review in some hospitals. In the *individuals* domain, IT- and IPC team experience in HAI surveillance was a key facilitator. Coordination center IT support helped address knowledge gaps, but implementation remained time-consuming when expertise was limited. Increasing experience in manual review boosted the confidence of the reviewers and expedited the manual part of the surveillance. Nurses found it easier to adhere to surveillance criteria, whereas some physicians struggled with personal clinical judgment interfering with the ECDC definition concept. For the *implementation process* domain, teamwork, task allocation, and milestone planning were crucial. Close collaboration between IT specialists and healthcare professionals ensured data completeness, while engagement with external experts and meticulous planning accelerated implementation. In the *outer setting* domain – e.g. other hospital departments, hospital management, IT infrastructure, and the broader healthcare environment – project alignment with hospital goals facilitated adoption. A well-established data warehouse with preprocessed data supported technical implementation, whereas the need for intensive data preparation (e.g., radiologic reports) and complex EHR structures slowed it down significantly. The manual review process was easier when all data were accessible within a single (compared to multiple) EHR system, and the hospital did not use paper-based documentation. In the *innovation* domain – referring to the semi-automated nvHAP surveillance system, including the computerized algorithm, materials, and the support from the coordination center team – time investment for manual review was the most important challenge for implementation. Manual review was considered challenging due to subjectivity in the interpretation of patient data, but training and support from the coordination center helped mitigate uncertainty. Well-designed project materials and ongoing technical support from the coordination center facilitated implementation.

## Discussion

In this feasibility study, seven Swiss acute care pilot hospitals successfully implemented a semi-automated, locally adaptable surveillance system for nvHAP. Sensitivity is the most important performance indicator in semi-automated surveillance with a proposed threshold of 90%.^13^ In the academic tertiary care centers and the public general hospitals, the implemented algorithms achieved sensitivities close to or at 100%, with lower 95% confidence interval margins consistently near or above 90%. Although the sample size in the community hospitals was too small to precisely quantify sensitivity, the available data and the algorithm’s design suggest that the algorithms likely performed comparably as in larger hospitals. Restricting the algorithm to indicators included as mandatory in the ECDC criteria implies that algorithms with fewer indicators may have equal or higher – but not lower – sensitivity. Thus, irrespective of the algorithm included, nvHAP cases could be missed by the algorithm as a result of technical implementation errors (e.g., nonconsideration of certain chest radiography types), text interpretation of radiology reports leading to erroneous exclusion of relevant chest images, or in rare instances of a pulmonary infiltrate being detected in a radiologic procedure not primarily targeting the chest (e.g., pneumonia detected in abdominal CT scan). The former was often the case when initially validating the performance of the algorithms during the technical implementation process, but meticulous overwork of input data finally led to sensitivities above 90%. This finding highlights the urgent need to validate an automated surveillance system after implementation and repetitively afterwards.

The second most important performance characteristic is the percentage of records to manually review, as it directly impacts the feasibility of manual nvHAP review in notoriously resource-constrained hospitals. A lower number of indicators included in the algorithm comes with the downside of a higher percentage of patients to manually review.^14^ This became evident in our project when comparing community hospitals 6 and 7, both using a four-indicator algorithm, which had a lower percentage of patients to manually review than hospital 5, which used one indicator only. Second, the patient population under surveillance plays an important role. Hospital 2 performed the study in two high-risk departments and although using a four-indicator algorithm, a high percentage of preselected patients had to be reviewed. The same algorithm resulted in a lower percentage of preselected patients in smaller hospitals, likely reflecting a lower-risk patient population with fewer diagnostic procedures performed.

When discussing feasibility of a semi-automated nvHAP surveillance, the time investment for technically implementing the algorithm is relevant. In our project, it ranged from 55 to 437 hours, with faster implementation observed in teams with prior experience with similar data-driven projects and access to preprocessed indicator variables. Qualitative interviews revealed that the need for detailed data preprocessing significantly slowed down the implementation process. The “onetime” investment to setup the computerized part of the surveillance is independent from hospital size and smaller hospitals often have limited IT resources. Thus, it likely is more difficult to absorb the upfront effort needed for tasks such as data extraction, verification, and algorithm implementation. On the other hand, manual review time is a critical factor for the sustained, long-term conduct of nvHAP surveillance. It is influenced by (1) the percentage of preselected records (see above), and (2) the time the pilot hospital team spends reviewing per record. In the pilot hospitals, the average time spent on one patient was 13.5 minutes. This is significantly longer than the 4 minutes reported by the coordination center team in 2017 reviewing USZ patients.^17^ This discrepancy may be due to the coordination center team’s figure representing a one-year average and the manual review conducted by three well-experienced team members, while pilot hospital teams reported data over six months, with work distributed among up to five first-time nvHAP reviewers. Qualitative interviews indicated that individuals with greater experience felt more confident and were able to expedite surveillance, suggesting that most differences in performance were attributable to limited surveillance practice. It is anticipated that manual review teams will significantly enhance their efficiency over time.

To our knowledge, this is the first study to evaluate the implementation and performance of a semi-automated nvHAP surveillance system across multiple acute care hospitals. While offering valuable insights to hospitals considering implementing such a surveillance, it has several limitations. First, although pilot hospitals were selected to reflect diverse hospital sizes, IT infrastructures, and linguistic regions, the sample does not fully capture the variability of hospitals within Switzerland. Conducted in a high-income country without smaller hospital teams implementing the algorithm, the study leaves open whether hospitals with fewer resources would be able to implement and conduct the surveillance. However, our findings suggest that even hospitals with minimal data infrastructure can operate semi-automated nvHAP surveillance based solely on radiologic procedures. Second, algorithm sensitivity was assessed using a six month validation period, resulting in unmet sample size goals. While lower 95% confidence interval limits were consistently near or above 90% in larger hospitals, the low number of nvHAP cases in community hospitals prevented accurate sensitivity quantification. Additionally, the validation cohort consisted of a preselected patient population of patients enriched for nvHAP risk (patients with ICD-10 HAP or hospital stay >2 weeks); thus, selection bias and overestimation of sensitivity cannot be excluded. Third, only two hospital teams conducted manual surveillance in five hospitals, limiting time assessment for manual review. Finally, interviews to identify implementation determinants were conducted by the coordinating team, introducing potential social desirability and observer bias. All efforts were made to foster trust between interviewer and interviewee, and to maintain reflexivity throughout data analysis to minimize these biases.

In conclusion, implementation and conduct of an nvHAP semi-automated surveillance proved to be feasible. The adaptability of the standard algorithm was important. Regardless of the algorithm implemented and the patient population under surveillance, algorithms were sufficiently sensitive and resulted in a significant reduction of workload for manual reviewers. Due to the very high sensitivity of the algorithm including 5 indicators, working with algorithms including fewer indicators will not relevantly affect comparability between hospitals, but will come with the disadvantage of higher workload for manual reviewers. As sufficient time must be allocated for the initial technical setup, validation, and manual review, implementation of semi-automated nvHAP surveillance can be particularly recommended for hospitals that have indicator data available in a preprocessed form and have IT and infection prevention teams experienced with similar projects.

## Supporting information

Wolfensberger et al. supplementary material Wolfensberger et al. supplementary material

## Supplementary material

To view supplementary material for this article, please visit https://doi.org/10.1017/ice.2026.10416.
