# Supplementary material for: Implementation of an adaptable semi-automated nonventilator hospital-acquired pneumonia (nvHAP) surveillance system in swiss acute care hospitals: a feasibility study
Source: Infect Control Hosp Epidemiol. 2026 Mar 26;47(5):441–7. doi: 10.1017/ice.2026.10416 (PMC13216788; doi:10.1017/ice.2026.10416)
Supplement: Wolfensberger et al. supplementary material [file S0899823X26104164sup001.pdf]

Appendix 1

Definition: Chest Radiology Procedure «of relevance»

Radiology is conducted >48h after admission (or anytime if re-admitted)

AND

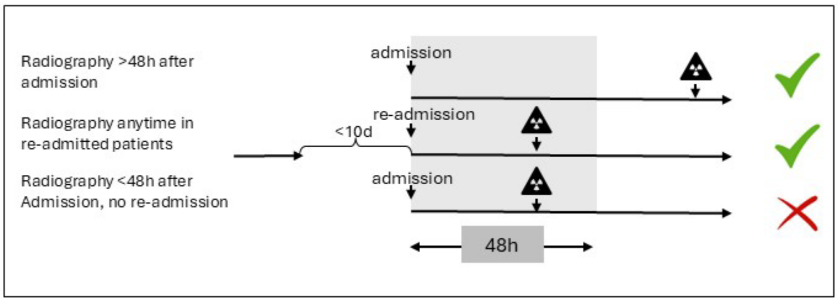

Radiology has no report that excludes pneumonia (text recognition, see Appendix)

AND

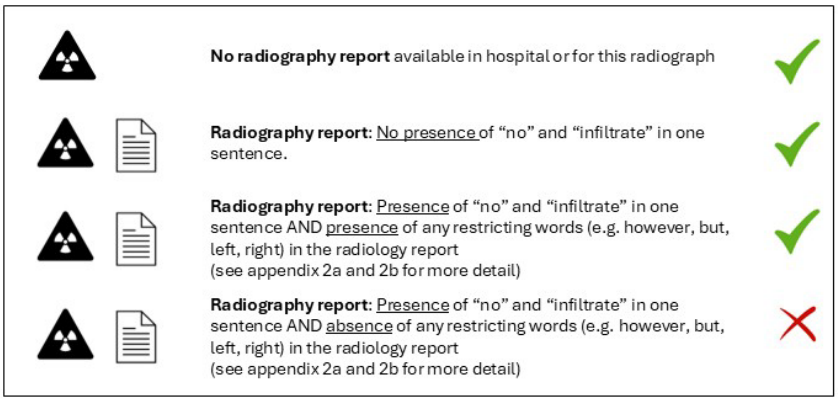

Radiology is in temporal relationship with leucopenia (<4G/l) or leucocytosis (≥12G/l) or fever (>38°C) (if data is available)

AND

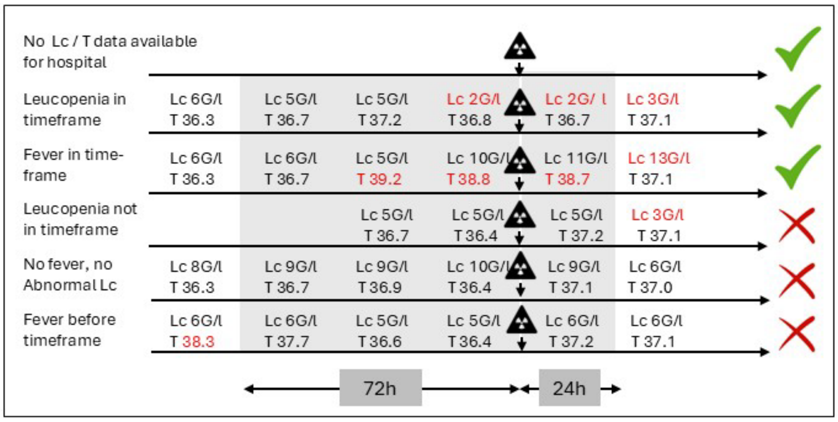

Radiology is not conducted after respiratory device permanently present for >48h (if data is available)

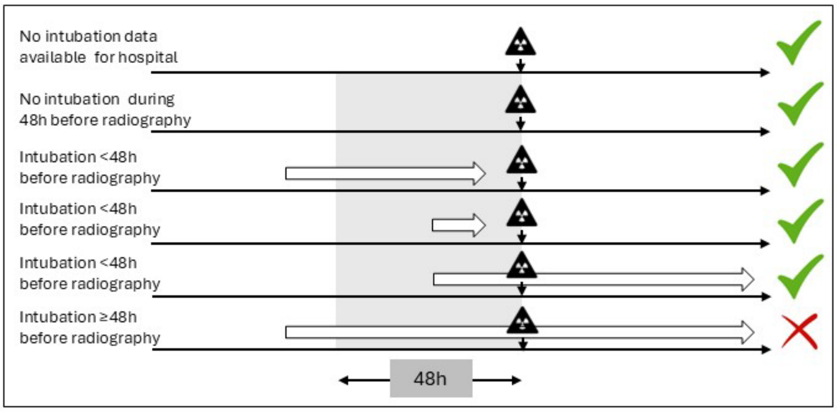

Chest Radiology procedure
Radiographic report
Intubation
Hospitalisation
Radiology of relevance
Radiology not of relevance

Appendix 2a. German version of full text analysis of radiologic procedures: Contradictory or restricting terms (with English translation)

Presence of the following two words in one sentence

“no” (German “kein”) and “infiltrate” (German “Infiltrat”)

AND absence of any of the following restricting words in the radiology report

**German**	**English**
“sonst”	otherwise
“ansonsten”	otherwise
“allerdings”	however
“jedoch”	however
“hingegen”	on the other hand
“aber”	but
“dafür”	but
“doch”	but
“obwohl”	although, though
“darüber hinaus”	furthermore, beyond that
“darüberhinaus”	furthermore
“dennoch”	nevertheless
“recht(s)*”	on the right (side), right, right sided…
“link(s)*”	on the left (side), left, left sided…
“ausser”	except
“ausgenommen”	except

Appendix 2b. French version of full text analysis of radiologic procedures: Contradictory or restricting terms (with English translation)

Presence of the following two words in one sentence

> “pas” and “pneumonie” OR “pas” and “infiltrat” OR “pas” and “foyer” OR “pas” and “infecti” OR “absence” and “pneumonie” OR “absence” and “infiltrat” OR “absence” and “foyer” OR “absence” and “infecti”

AND Absence of any of the following restricting word in the report

> “autre”, “mais”, “par contre”, “cependant”, “sinon”, “sauf”, “à l’exception”, “hormis”, “outre”, "néanmions ", “exclu”

Appendix 3. Definition criteria for Hospital-acquired Pneumonia (HAP), specifically nvHAP and iHAP (non-ventilator-associated HAP and indeterminate HAP) for adult patients (>16years)

General definition HAP

- Hospital-acquired pneumonia occurs when the first symptoms or signs of pneumonia appear more than 48 h after admission or up to 48 h after hospital discharge.
- Only pneumonia acquired in the monitoring hospital are considered relevant for the current surveillance. Also, the definition criteria apply only to adult patients.
- Depending on the presence of a respiratory device (such as tube, tracheostoma) prior to the onset of the first symptoms, HAP is classified as nvHAP (non-ventilator-associated pneumonia), VAP (ventilator-associated pneumonia) or iHAP (indeterminate HAP).

Specific definition of HAP

To diagnose HAP, the following criteria must apply:

Radiology[1]**:**

Two or more serial chest X-rays or CT-scans with a suggestive image of pneumonia[2] for patients with underlying cardiac or pulmonary disease **OR**

One definitive chest X-ray or CT-scan with a suggestive image of pneumonia is sufficient in patients without underlying cardiac or pulmonary disease or in patients with underlying cardiac or pulmonary disease if comparison with previous X-rays is possible (image not older than 1 year)

AND at least one of the following:

- fever > 38 °C with no other cause;
- leukopenia (<4 000 WBC/mm^3)
- leucocytosis (≥12 000 WBC/mm^3)

AND at least one of the following (or at least two if clinical pneumonia only = PN 4 and PN 5 (see below))

- new onset of purulent sputum [3] or change in character of sputum (colour, odour, quantity, consistency)
- new onset of cough or dyspnea or tachypnea [4]
- new onset of suggestive auscultation (rales or bronchial breath sounds), ronchi, wheezing

---

[1] In patients with pulmonary or cardiac disease (for example: interstitial lung disease or congestive heart failure), the diagnosis of pneumonia may be particularly difficult. For example: Pulmonary edema from decompensated congestive heart failure may simulate the presentation of pneumonia. To help confirm difficult cases, multiple imaging test results spanning over several calendar days must be considered when determining if there is imaging test evidence of pneumonia. Pneumonia may have rapid onset and progression but does not resolve quickly. Imaging test evidence of pneumonia will persist. Rapid imaging resolution suggests that the patient does not have pneumonia, but rather a non-infectious process such as atelectasis or congestive heart failure (Ref: CDC)

[2] With at least one of the following: new and persistent ***or*** progressive and persistent infiltrate, consolidation, cavitation

[3] If sputum is examined in the microbiology lab, purulent sputum is defined as secretions from the lungs, bronchi, or trachea that contain ≥25 neutrophils and ≤10 squamous epithelial cells per low power field (x100). If your laboratory reports these data semi-quantitatively or uses a different format for reporting Gram stain or direct examination results contact the laboratory to get information about what quantitative ranges the semi-quantitative report corresponds to. If the laboratory cannot provide additional information use the following direct examination results to meet the purulent respiratory secretions criterion: many, heavy, numerous 4+, or ≥25 neutrophils per low power field (lpf) [x100], AND no, rare, occasional, few, 1+ or 2+, or ≤10 squamous epithelial cells per lpf [x100]. (according to CDC). If sputum was not sent for examination, refer to written clinical description.

[4] >25/min (according to CDC)

- worsening gas exchange (e.g. O2 desaturation or increased oxygen requirements or increased ventilation demand)

AND according to the used diagnostic method [5]:

a) Bacteriologic diagnostic test performed by[6]:
    - Positive quantitative culture[7] from minimally contaminated LRT (lower respiratory tract) specimen **(PN 1)**
        - broncho-alveolar lavage (BAL) with a threshold of > 10^4 CFU2/ml or >5 % of BAL obtained cells contain intracellular bacteria on direct microscopic exam (classified on the diagnostic category BAL);
        - protected brush (PB Wimberley) with a threshold of >10^3 CFU/ml;
        - distal protected aspirate (DPA) with a threshold of >10^3 CFU/ml.
    - Positive quantitative culture from possibly contaminated LRT specimen **(PN 2)**
        - Quantitative culture of LRT specimen (e.g. endotracheal aspirate) with a threshold of 106 CFU/ml [8]

b) Alternative microbiology methods **(PN 3)**
    - positive blood culture[9] not related to another source of infection
    - Positive growth in culture of pleural fluid
    - pleural or pulmonary abscess with positive needle aspiration
    - histologic pulmonary exam shows evidence of pneumonia
    - positive exams for pneumonia with virus or particular germs (Legionella, Aspergillus, mycobacteria, mycoplasma, Pneumocystis carinii):
        - o positive detection of viral antigen or antibody from respiratory secretions (e.g. EIA, FAMA, shell vial assay, PCR);
        - o positive direct exam or positive culture from bronchial secretions or tissue;
        - o seroconversion (e.g. influenza viruses, Legionella, Chlamydia);
        - o detection of antigens in urine (Legionella)

c) positive sputum culture or non-quantitative LRT specimen culture or quantitative LRT with < 106 CFU/ml **(PN 4)**

d) no positive microbiology **(PN 5)**

---

[5] There is a hierarchy of specific categories within the major site pneumonia. If the patient meets criteria for more than one subcategory, categorize it as the lowest number (i.e. PN2 rather than PN3).

[6] If the Microbiology laboratory delivers semi-quantitative (e.g. few, many…) and not quantitative results, consult with your laboratory to determine how reported semi-quantitative results match the quantitative thresholds.

[7] Coagulase-negative *Staphylococcus* species, *Enterococcus* species and *Candida* species are frequent colonizators and can only be used to meet PN definitions when identified from pleural fluid obtained during thoracentesis or initial placement of chest tube (not from an indwelling chest tube) or lung tissue.

[8] Or highest category according to laboratory performing the testing.

[9] Any coagulase-negative *Staphylococcus* species, *Enterococcus* species and *Candida* species or yeast not otherwise specified that are identified from blood cannot be deemed secondary to a PN, unless the organism was also identified from pleural fluid (where specimen was obtained during thoracentesis or initial placement of chest tube and NOT from an indwelling chest tube) or lung tissue (or for Candida species: identification from sputum or LRT specimen) (CDC).

Distinction between nvHAP and iHAP

NvHAP (non-ventilator-associated hospital acquired pneumonia): if the above mentioned criteria are met and there was no respiratory device present in the 48 hours preceding the onset of infection, the pneumonia is considered an nvHAP.

iHAP (indeterminate hospital acquired pneumonia): if the above mentioned criteria are met and a respiratory device was intermittently present in the 48 hours preceding the onset of infection, the pneumonia is considered an iHAP.

NvHAP in patients with healthcare- associated COVID-19

COVID-19 pneumonia are coded as nvHAP if the patient fulfils the above mentioned definition criteria AND fulfills definition criteria of probable or definite healthcare-associated COVID-19 (see ECDC criteria below).

- Probable healthcare-associated COVID-19 (HA-COVID-19):
    - Symptoms onset on day 8-14 after admission
    - Symptom onset on day 3-7 and a strong suspicion of healthcare transmission.
- Definite HA-COVID-19:
    - Symptom onset on day >14 after admission

NvHAP in patients with community-acquired COVID-19

To diagnose nvHAP in patients with community-acquired COVID-19 is challenging. In principle, the above mentioned diagnostic nvHAP criteria have to be fulfilled, but the natural course of COVID-19 has to be considered. A continuous deterioration of oxygen saturation and/or an increase of pulmonary infiltrates can be encountered in the natural course of COVID-19. To diagnose nvHAP the pulmonary infiltrates have to be progressive and there has to be a new or worsening leukocytosis/leucopenia or new fever (i.e. the persistence of these signs/symptoms is not sufficient). Manifestations pointing to possible nvHAP in patients with COVID-19 can for example be: worsening of clinical symptoms after initial improvement, lobar infiltrate, purulent sputum, or new detection of microbiologic relevant pathogen.

Fungal pneumonia

Fungal pneumonia is defined as "possible fungal pneumonia" according to EORTC criteria, i.e. cases meeting the criteria for a host factor and a clinical criterion [23].

Definition criteria fungal pneumonia EORTC:

Possible 1 host factor AND 1 clinical criterion.

Probable 1 host factor, 1 clinical criterion AND 1 mycological criterion.

Proven Histopathological findings or culture from sterile environment.

Host factors:

- Recent history of neutropenia (10 days) temporally related to the onset of invasive fungal disease
- Hematologic malignancy
- Receipt of an allogeneic stem cell transplant
- Receipt of a solid organ transplant
- Prolonged use of corticosteroids (excluding among patients with allergic bronchopulmonary aspergillosis) at a therapeutic dose of ≥0.3 mg/kg corticosteroids for ≥3 weeks in the past 60 days

- Treatment with other recognized T-cell immunosuppressants, such as calcineurin inhibitors, tumor necrosis factor-a blockers, lymphocyte-specific monoclonal antibodies, immunosuppressive nucleoside analogues during the past 90 days
- Treatment with recognized B-cell immunosuppressants, such as Bruton's tyrosine kinase inhibitors, eg, ibrutinib
- Inherited severe immunodeficiency (such as chronic granulomatous disease, STAT 3 deficiency, or severe combined immunodeficiency)
- Acute graft-versus-host disease grade III or IV involving the gut, lungs, or liver that is refractory to first-line treatment with steroids

Clinical features of pulmonary aspergillosis with the presence of 1 of the following 4 patterns on CT

- Dense, well-circumscribed lesions(s) with or without a halo sign
- Air crescent sign
- Cavity
- Wedge-shaped and segmental or lobar consolidation

Appendix 4. Interview Guides, Baseline and Conclusion

Interview at Project Start

To be filled in by interviewer	
Who (Name, hospital, function/professional group)	
When (Date)	
Consent audio?	
Impression?	

Question	Notes	Insights
**Please introduce yourself briefly and describe your role in the project**		
**Please describe the "Team CHAPS" at your pilot hospital?**<br>**What makes your team special?**		
Who do you think is interested in setting up an nvHAP surveillance in your hospital? (team leads)		
**Could you share your overall impression of the CHAPS project?**<br>○ What is working well?<br>○ Where do you see challenges?		
**Are the CHAPS project materials (handbook, workflow, data entry form, etc.) helpful to you?**<br>○ Where did you have to make adjustments?		
**Is there anything else you would like to add?**		

Interview at Project End

To be filled in by interviewer	
Who (Name, hospital, function/professional group)	
When (Date)	
Consent audio?	
Impression?	

Question	Notes	Follow-up questions from baseline interview
**What specific tasks or responsibilities have you handled in this project?**		

**Could you share your overall impression of the CHAPS project**<br><br>o What did you like / find easy?<br><br>o Where did you encounter challenges?		
**Did you need to make any adjustments during the project?**<br><br>o Which ones?		
**How would you describe the support you received from the coordination center team?**<br><br>(Handbook, teaching materials, EXCEL data entry form, etc.)		
**If you were to start the project again, what would you do differently?**		
**Is there anything else you would like to add?**		
